# Supplementary material for: Anterior middle cingulate cortex gamma-aminobutyric acid level is elevated in children with both familial and prenatal alcohol exposure-associated attention deficit hyperactivity disorder
Source: medRxiv. 2026 May 26:2026.05.25.26354065. Preprint. [Version 1] doi: 10.64898/2026.05.25.26354065 (PMC13232367; doi:10.64898/2026.05.25.26354065)
Supplement: Supplementary file 1 [file NIHPP2026.05.25.26354065v1-supplement-1.pdf]

medRxiv preprint doi: https://doi.org/10.64898/2026.05.25.26354065; this version posted May 26, 2026. The copyright holder for this preprint (which was not certified by peer review) is the author/funder, who has granted medRxiv a license to display the preprint in perpetuity.
It is made available under a CC-BY 4.0 International license .

SUPPLEMENTAL MATERIALS

Table S1. MRSinMRS checklist.

Site (Name or Number)			
**1. Hardware**			
a. Field strength [T]	3 T		
b. Manufacturer	Siemens		
c. Model (software version if available)	Prisma VE11C		
d. RF coils: nuclei (transmit/ receive), number of channels, type, body part	proton, 32 channels, phased-array, head		
e. Additional hardware	none		
**2. Acquisition**			
a. Pulse sequence	Siemens 839G WIP<br>J-difference-edited single-voxel MRS		
b. Volume of Interest (VOI) locations	midline (left+right) anterior middle cingulate cortex		
c. Nominal VOI size [cm^3, mm^3]	4.0 cm (anterior-posterior) x 3.0 cm (left-right) x 1.0 cm (superior-inferior) = 12.0 cc		
d. Repetition Time (TR), Echo Time (TE) [ms, s]	2000/68 ms		
e. Total number of Excitations or acquisitions per spectrum<br>In time series for kinetic studies<br>i. Number of Averaged spectra (NA) per time-point<br>ii. Averaging method (e.g. block-wise or moving average)<br>iii. Total number of spectra (acquired / in time-series)	128 Invert/128 Control		

medRxiv preprint doi: https://doi.org/10.64898/2026.05.25.26354065; this version posted May 26, 2026. The copyright holder for this preprint
(which was not certified by peer review) is the author/funder, who has granted medRxiv a license to display the preprint in perpetuity.
It is made available under a CC-BY 4.0 International license .

f. Additional sequence parameters<br><br>(spectral width in Hz, number of spectral points, frequency offsets)<br><br>If STEAM:, Mixing Time (TM)<br><br>If MRSI: 2D or 3D, FOV in all directions, matrix size, acceleration factors, sampling method	1200 Hz, 1024 spectral points, delta -2.8 ppm<br><br>edit pulse frequency 1.90 ppm<br><br>edit center frequency 4.70 ppm		
g. Water Suppression Method	CHESS		
h. Shimming Method, reference peak, and thresholds for “acceptance of shim” chosen	Siemens Brain shim		
i. Triggering or motion correction method<br><br>(respiratory, peripheral, cardiac triggering, incl. device used and delays)	none		
**3. Data analysis methods and outputs**			
a. Analysis software	SVFit2016		
b. Processing steps deviating from quoted reference or product	none		
c. Output measure<br><br>(e.g. absolute concentration, institutional units, ratio)Processing steps deviating from quoted reference or product	Institutional Units, ratios		
d. Quantification references and assumptions, fitting model assumptions	water reference		
**4. Data Quality**			
a. Reported variables<br><br>(SNR, Linewidth (with reference peaks))	SNR, linewidth of Cr 3.03 ppm peak		
b. Data exclusion criteria	SNR<5, Linewidth>0.03<br><br>voxel GM < 40%		

medRxiv preprint doi: https://doi.org/10.64898/2026.05.25.26354065; this version posted May 26, 2026. The copyright holder for this preprint (which was not certified by peer review) is the author/funder, who has granted medRxiv a license to display the preprint in perpetuity.
It is made available under a CC-BY 4.0 International license .

	voxel CSF > 15%		
c. Quality measures of postprocessing Model fitting (e.g. CRLB, goodness of fit, SD of residual)	CRLB		
d. Sample Spectrum	included		
